# Macrophage migration inhibitory factor is involved in antineutrophil cytoplasmic antibody-mediated activation of C5a-primed neutrophils

**DOI:** 10.1186/s12865-019-0306-z

**Published:** 2019-06-27

**Authors:** Jian Hao, Tiegang Lv, Liping Xu, Mao Ran, Kaili Wu

**Affiliations:** Renal Division, Department of Medicine, the Affiliated Hospital of Inner Mongolia Medical University, Huhehot, 010050 Inner Mongolia China

**Keywords:** Antineutrophil cytoplasmic antibody, C5a, Degranulation, Macrophage migration inhibitory factor, Neutrophil, Respiratory burst

## Abstract

**Background:**

C5a is important for antineutrophil cytoplasmic antibody (ANCA)-mediated activation of neutrophils. The present study aimed to assess the role of macrophage migration inhibitory factor (MIF) in ANCA-mediated activation of C5a-primed neutrophils. The effects of MIF on ANCA-mediated neutrophil respiratory burst and degranulation were determined. In addition, the effect of a MIF antagonist on the activation of C5a-primed neutrophils was assessed.

**Results:**

MIF treatment resulted in increased membrane proteinase-3 (mPR3) expression on neutrophils and enhanced myeloperoxidase (MPO) amounts in neutrophil culture supernatants. The concentration of MIF was significantly higher in the neutrophils supernatant primed with C5a (negative control: 14.2 ± 1.16 ng/ml; C5a: 45.8 ± 2.8 ng/ml, *P* < 0.001 vs. negative control; C5a + IgG: 44.8 ± 1.93 ng/ml, P < 0.001 vs. negative control; C5a + MPO-ANCA: 73.0 ± 5.5 ng/ml, P < 0.001 vs. C5a; and C5a + PR3-ANCA: 69.4 ± 5.35 ng/ml, P < 0.001 vs. C5a). MIF primed neutrophils to undergo respiratory burst and degranulation in response to ANCA. Indeed, mean fluorescence intensity (a measure of respiratory burst) was significantly higher in MIF-primed neutrophils activated with MPO-ANCA-positive IgG or PR3-ANCA-positive IgG compared with non-primed neutrophils. Meanwhile, a MIF antagonist reduced oxygen radical production in C5a-primed neutrophils treated with patient-derived ANCA-positive IgG.

**Conclusions:**

MIF can prime neutrophils to undergo ANCA-mediated respiratory burst and degranulation. Blocking MIF resulted in reduced ANCA-mediated activation of C5a-primed neutrophils. These findings indicated that the interaction between MIF and C5a may contribute to ANCA-mediated neutrophil activation.

## Background

Granulomatosis with polyangiitis (GPA, or Wegener’s granulomatosis), microscopic polyangiitis (MPA) and eosinophilic granulomatosis with polyangiitis (EGPA, or Churg-Strauss syndrome) are systemic diseases affecting small vessels featuring vessel wall segmentation with necrotizing inflammation and immunoglobulin deposition [1, 2]. GPA, MPA and EGPA are considered subsets of antineutrophil cytoplasmic antibody (ANCA)-associated vasculitis (AAV) due to their association with the presence of ANCAs [1, 2]. Immunoglobulin G (IgG) autoantibodies against cytoplasmic components of neutrophils, including proteinase-3 (PR3) and myeloperoxidase (MPO), represent characteristic ANCAs [3].

The current hypothesis is that AAV pathogenesis involves neutrophils. Indeed, these cells constitute the first line of defense against bacteria and fungi. Neutrophils kill target cells using a combination of phagocytosis, oxygen radicals produced through respiratory burst, and the release of intracellular granules containing antimicrobial and inflammatory factors (degranulation) [4]. Lactoferrin is one of the iron-binding glycoproteins found in neutrophil granules, and can be used as a marker of neutrophil degranulation [5, 6]. Previous preclinical studies showed that ANCAs are able to stimulate respiratory burst and degranulation in primed neutrophils. The above effects are considered important contributors to the development of AAVs [7–11]. Indeed, the pathogenic potential of ANCAs has been demonstrated in a mouse model of vasculitis induced by anti-MPO antibodies [12]. Furthermore, animal studies demonstrated that neutrophils are the main cellular effectors in AAVs [12, 13]. Nevertheless, previous clinical and in vivo studies have implicated different pathways in the pathogenesis of AAVs [14–18]. It was shown that priming of neutrophils by C5a is necessary for an ANCA-induced respiratory burst to occur [19]. Although therapies targeting C5a have shown some initial promise [20], our knowledge of the key molecules and physiological events activating C5a-primed neutrophils by ANCAs is still limited at best.

Macrophage migration inhibitory factor (MIF) represents a proinflammatory cytokine with important roles in the innate and adaptive immune systems [21]. The cytoplasm of human nucleated cells contains MIF, which is released in response to cellular stress, leading to the activation of the CD74/CD44 receptor complex [21–24]. Hence, it was hypothesized that MIF plays important roles in a number of diseases, including chronic renal disease [25], asthma [26], rheumatoid arthritis [27], inflammatory bowel diseases [28, 29], systemic lupus erythematosus [30, 31] and crescentic glomerulonephritis [30]. MIF enhances the chemotactic responses and survival of neutrophils [32, 33], and is therefore involved in AAVs [34, 35]. C5a stimulates neutrophils to produce MIF during sepsis, and C5a receptor inhibition decreases the release of MIF during the initial stages of sepsis [36].

It was previously reported that active AAV cases have high plasma levels of MIF, while MIF-primed neutrophils could be stimulated by MPO-ANCA-positive IgG or PR3-ANCA-positive IgG to undergo respiratory burst and degranulation [37]. Nevertheless, MIF’s function in C5a-primed neutrophils remains unknown. Based on the above findings, we hypothesized that MIF released from C5a-primed neutrophils would boost neutrophil activation and contribute to ANCA-induced respiratory burst and degranulation in these cells. Therefore, the present study assessed the effect of MIF on ANCA-associated activation of C5a-primed neutrophils.

## Materials and methods

### IgG preparation

Cases of active MPO-ANCA- or PR3-ANCA-positive primary AAV (none had both ailments) were recruited, and ANCA-positive IgG molecules were extracted from their plasma [38, 39]. Healthy volunteers were recruited, and normal IgG was obtained [40, 41]. We controlled the amount of ANCA-positive IgG (total IgG), and the amount of ANCA-IgG was kept consistent for each individual patient in the experiment. LPS amounts in ANCA-positive IgG samples were < 0.1 ng/ml.

### Neutrophil isolation

Neutrophils from heparin-treated venous blood obtained from healthy individuals were isolated by density gradient centrifugation using Lymphoprep (Nycomed, Norway). Red blood cell lysis was performed with chilled NH_4_Cl buffer, and neutrophils (purity > 95%) underwent washing with Ca^2+^/Mg^2+^-free Hanks balanced salt solution (HBSS−/−; Chemical Reagents, China) and resuspended at 2.5 × 10^7^ cells/ml in HBSS containing calcium and magnesium (HBSS+/+; Chemical Reagents). This suspension was used in subsequent experiments [40].

This study followed the Declaration of Helsinki, and had approval from the Clinical Research Ethics Committee of the Affiliated Hospital of Inner Mongolia Medical College. Each participant or donor provided written informed consent.

### Membrane expression of PR3 and MPO in neutrophils

PR3 and MPO levels on the neutrophil surface were examined by flow cytometry. C5a (100 ng/ml for 15 min at 37 °C; Biovision, USA) primed and control neutrophils were administered MIF (50 ng/ml; Sigma-Aldrich, USA) or not (buffer without MIF) for 30 min at 37 °C. All subsequent procedures were carried out on ice; all washes were performed with HBSS+/+ containing 1% bovine serum albumin (BSA). Heat-denatured goat IgG (0.5 mg/ml) was incubated with the cells for 15 min for Fcγ receptor saturation. The neutrophils underwent staining for 30 min with mouse monoclonal IgG1 antibodies targeting human PR3 (WGM2; Abcam, UK) and MPO (2C7; Abcam), respectively, or control IgG1 antibodies (BioLegend, USA). This was followed by incubation with phycoerythrin (PE)-conjugated goat anti-mouse antibodies (Abcam) and 0.5 mg/ml heat-treated goat IgG. The expression levels of ANCA antigens were evaluated flow-cytometrically based on PE fluorescence on a BD FACScan (Becton Dickinson, USA). A total of 10,000 cells were analyzed per specimen. PR3 and MPO levels were assessed as mean fluorescence intensity (MFI) values of specific signals. In certain experiments, neutrophils underwent pre-treatment with the small-molecule MIF antagonist (S,R)3-(4-hydroxyphenyl)-4,5-dihydro-5-isoxazole acetic acid methyl ester (ISO-1; Tocris Bioscience, USA) [42, 43] or vehicle (dimethyl sulfoxide, DMSO) prior to other interventions [44].

### Measurement of MPO levels in cell culture supernatants

A commercial enzyme-linked immunosorbent assay (ELISA) (USCNK, China) kit was used for measuring MPO levels in the supernatants of neutrophils treated with MIF and/or C5a, as directed by the manufacturer. Absorbance was measured at 450 nm on a spectrophotometer.

### Measurement of MIF levels in cell culture supernatants

A commercial kit (Abcam, UK) was used for measuring MIF levels in the supernatant of neutrophil cultures treated with C5a (100 ng/ml) and ANCA-positive IgG or buffer, according to the manufacturer’s instructions. The absorbance was measured at 450 nm.

### Respiratory burst assessment

The production of reactive oxygen species was estimated based on dihydrorhodamine (DHR, non-fluorescent) oxidation into rhodamine (green fluorescence). Neutrophils were administered 0.05 mM DHR123 (Sigma-Aldrich) for 10 min at 37 °C, with 2 mM NaN_3_ supplemented for preventing intracellular degradation of H_2_O_2_. Then, the cells were sequentially administered MIF (50 ng/ml) or C5a (100 ng/ml) [45], and normal or patient-derived ANCA-positive IgG (200 μg/ml) for 1 h at 37 °C. Reactions were terminated by addition of 1 ml of ice-cold HBSS containing 1% BSA. Data (10,000 cells per sample) were collected by flow cytometry on a BD FACScan. MFI values determined for all experimental conditions represented the amounts of reactive oxygen species produced [40, 41].

### ANCA-induced degranulation

Neutrophils treated with MIF and/or C5a were administered MPO-ANCA-positive, PR3-ANCA-positive, or normal IgG for 1 h, with control cells treated with the buffer. Some groups of cells also underwent pre-treatment with ISO-1 or DMSO (15 min on ice). Supernatant lactoferrin content was detected with an ELISA kit (USCNK, China), as directed by the manufacturer.

### Statistical analysis

The Shapiro-Wilk test was employed to assess data normality. Quantitative data are mean ± standard deviation (SD) (normal distribution) or median and range (skewed distribution). Student’s t test and the Mann-Whitney U test were used to compare data with normal and non-normal distributions, respectively. *P* < 0.05 indicated statistical significance. All analyses were carried out with SPSS 16.0 (SPSS, USA).

## Results

### MIF increases mPR3 levels on neutrophils and MPO amounts in culture supernatants

**Fig. 1:**
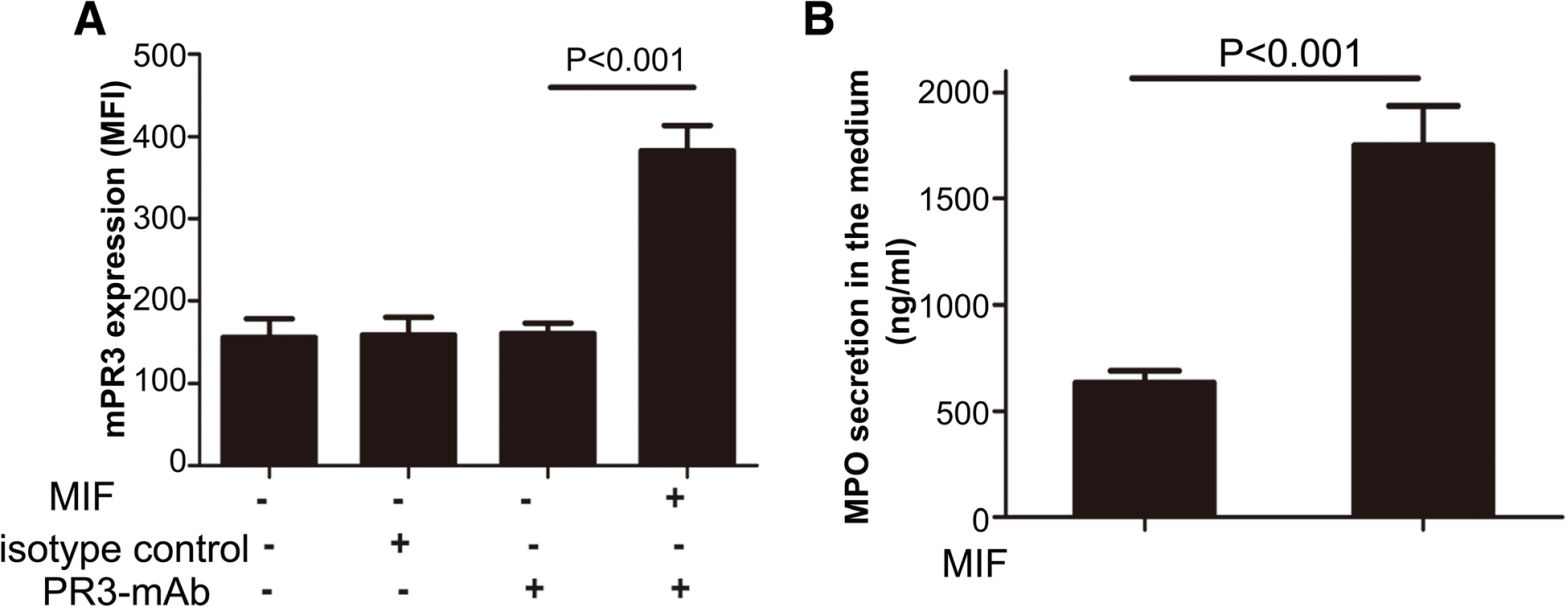
Macrophage migration inhibitory factor (MIF) enhances the translocation of antineutrophil cytoplasmic antibody (ANCA) antigens. **a** Expression of membrane proteinase-3 (mPR3) on neutrophils obtained from five healthy donors. mAb, monoclonal antibody; MFI, mean fluorescence intensity. **b** Myeloperoxidase (MPO) levels in culture supernatants of neutrophils obtained from five healthy donors. Bars represent mean ± standard deviation of measurements made in five independent experiments

Neutrophil expression of mPR3 and MPO levels in cell supernatants were measured using neutrophils obtained from five healthy individuals, administered 0 and 50 ng/ml MIF, respectively. The mPR3 expression levels were markedly elevated in MIF-primed neutrophils compared with control cells (382.8 ± 13.8 vs. 156.2 ± 9.98, *P* < 0.001) (Fig. 1a). Furthermore, MPO amounts were starkly higher in supernatants from MIF-primed neutrophils compared with the control value (1752.1 ± 83.7 ng/ml vs. 632.8 ± 25.27 ng/ml, *P* < 0.001) (Fig. 1b).

### ANCA stimulates MIF-treated neutrophils to undergo respiratory burst and degranulation

**Fig. 2:**
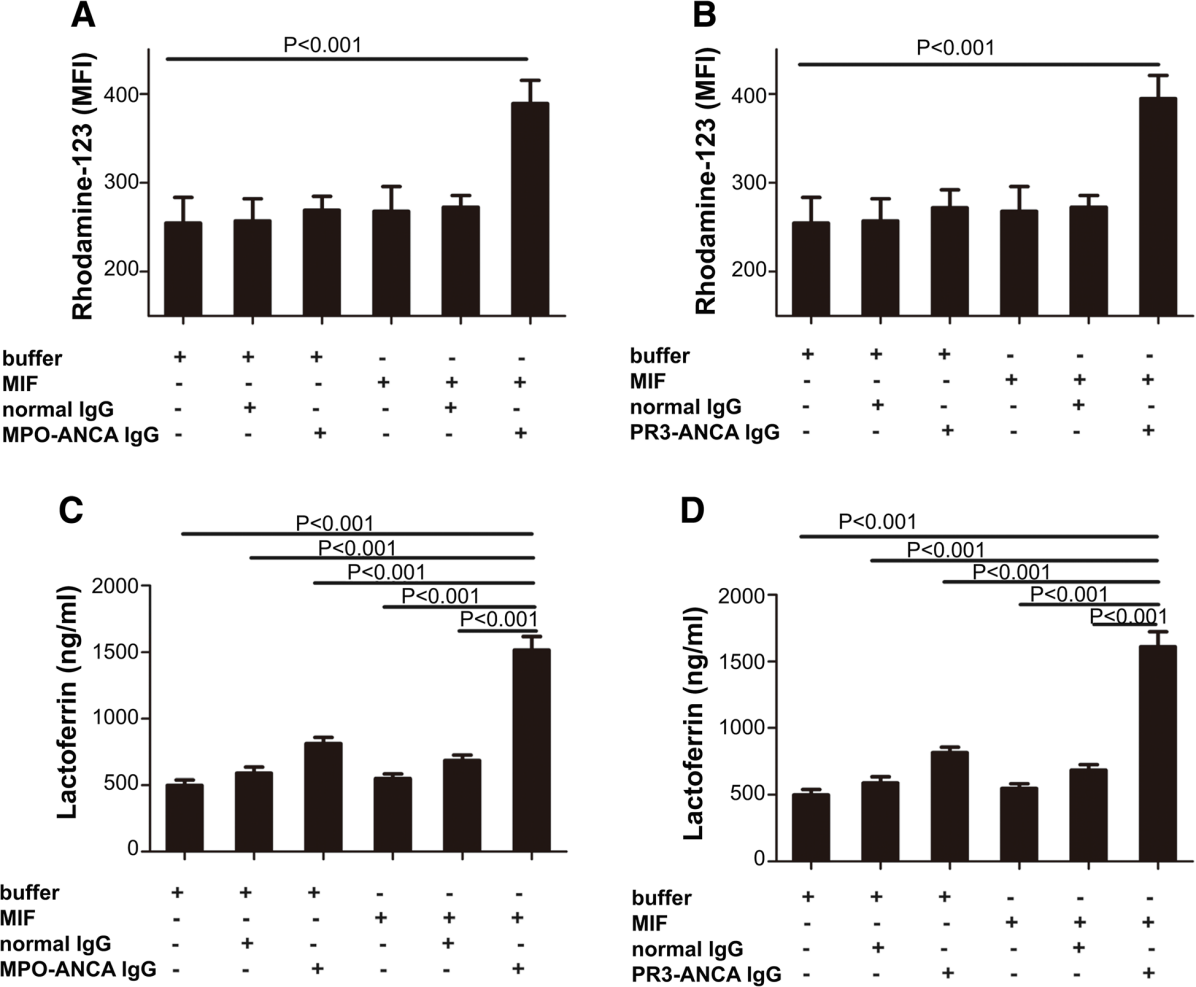
Respiratory burst and degranulation in macrophage migration inhibitory factor (MIF)-primed neutrophils induced by membrane proteinase-3 (PR3)-antineutrophil cytoplasmic antibody (ANCA)-positive immunoglobulin G (IgG) or myeloperoxidase (MPO)-ANCA-positive IgG. Respiratory burst induced by patient-derived MPO-ANCA-positive IgG (**a**) or PR3-ANCA-positive IgG (**b**) in MIF-primed and non-primed neutrophils obtained from five healthy donors, respectively, was reflected by dihydrorhodamine conversion into rhodamine-123. MFI, mean fluorescence intensity. Neutrophil degranulation induced by patient-derived MPO-ANCA-positive IgG (**c**) or PR3-ANCA-positive IgG (**d**) was measured in MIF-primed and non-primed neutrophils obtained from five healthy donors. Bars represent mean ± standard deviation of measurements made in five independent experiments

ANCA-positive IgGs (obtained from 5 active MPO-ANCA- and 3 active PR3-ANCA-positive vasculitis cases) were used to assess whether ANCA would induce respiratory burst in MIF-treated neutrophils. MIF was used at 50 ng/ml, which increased the expression levels of mPR3 on neutrophils. In comparison with untreated cells, MIF-primed neutrophils administered MPO-ANCA-positive IgG (389.4 ± 11.72 vs. 254.8 ± 12.8, *P* < 0.001) or PR3-ANCA-positive IgG (394.8 ± 11.71 vs. 254.8 ± 12.8, *P* < 0.001) showed markedly elevated MFI values (Fig. 2a and b). Meanwhile, MIF, ANCA-IgG, or normal IgG caused no overt respiratory burst, and neither did MIF combined with normal IgG.

Degranulation levels were evaluated by determination of lactoferrin amounts in supernatants from neutrophils treated with MIF and ANCA-positive IgGs. In comparison with unstimulated cells, MIF-treated neutrophils exposed to MPO-ANCA-positive IgG (1515.0 ± 44.94 ng/ml vs. 499.2 ± 18.64 ng/ml, *P* < 0.001) or PR3-ANCA-positive IgG (1613.0 ± 49.52 ng/ml vs. 499.2 ± 18.64 ng/ml, *P* < 0.001) showed markedly increased lactoferrin amounts in supernatants. Although degranulation was somewhat stimulated in neutrophils administered ANCA-IgG only, normal IgG only or MIF combined with normal IgG, further significant increases were observed following exposure to MPO-ANCA- or MPO-ANCA-positive IgG. In contrast, degranulation levels did not differ between neutrophils treated with normal IgG alone and cells administered MIF combined with normal IgG (Fig. 2c and d).

### MIF levels in the supernatants of C5a-primed neutrophil

**Fig. 3:**
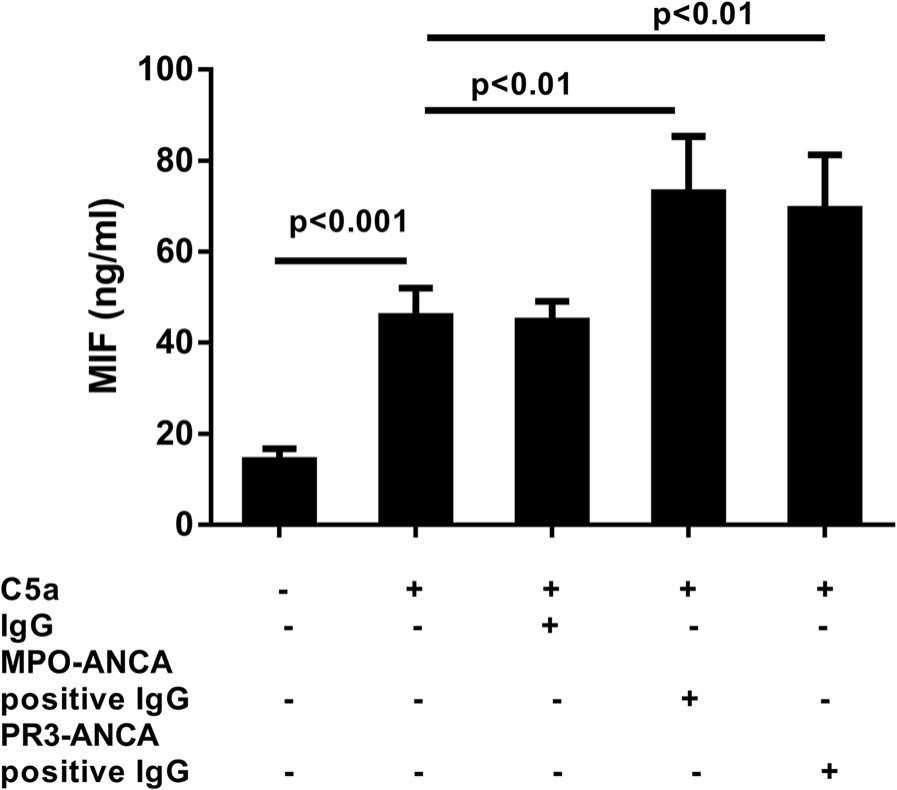
Macrophage migration inhibitory factor (MIF) levels in the C5a-primed neutrophils supernatants and administered antineutrophil cytoplasmic antibody (ANCA). MIF levels in the supernatant of neutrophils (obtained from five healthy donors) under various experimental conditions measured using a commercial kit (Abcam, UK). C5a-supernatant, supernatant from neutrophils primed with C5a; MPO-ANCA-positive IgG, patient-derived myeloperoxidase-ANCA-positive immunoglobulin G; PR3-ANCA-positive IgG, patient-derived membrane proteinase-3-ANCA-positive immunoglobulin G. Bars represent mean ± standard deviation of measurements made in 5 independent experiments and donors

Neutrophils were incubated with C5a (100 ng/L) and MIF concentration in the supernatant was detected by ELISA. The concentration of MIF was significantly higher in the neutrophils supernatant primed with C5a (negative control: 14.2 ± 1.16; C5a: 45.8 ± 2.8, *P* < 0.001 vs. negative control; C5a + IgG: 44.8 ± 1.93, P < 0.001 vs. negative control; C5a + MPO-ANCA: 73.0 ± 5.5, P < 0.001 vs. C5a; and C5a + PR3-ANCA: 69.4 ± 5.35 ng/ml, P < 0.001 vs. C5a) (Fig. 3).

### A MIF antagonist inhibits ANCA-associated respiratory burst and degranulation in C5a-treated neutrophils

**Fig. 4:**
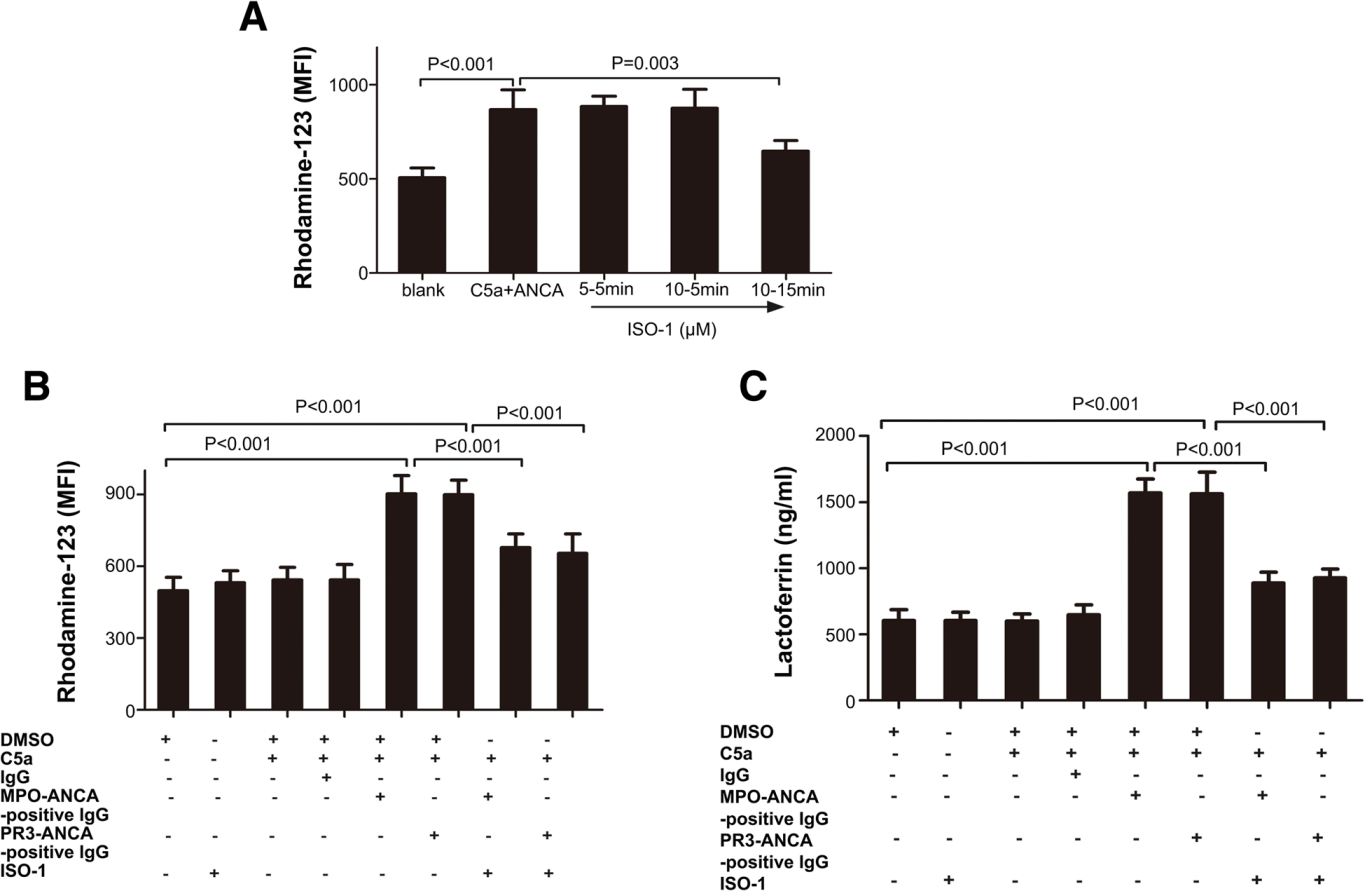
A macrophage migration inhibitory factor (MIF) antagonist inhibits antineutrophil cytoplasmic antibody (ANCA)-induced respiratory burst and degranulation in C5a-primed neutrophils. **a** Neutrophil respiratory burst was reflected by dihydrorhodamine-123 conversion into rhodamine-123. Neutrophils obtained from five healthy donors were pre-incubated with various concentrations of (S,R)3-(4-hydroxyphenyl)-4,5-dihydro-5-isoxazole acetic acid methyl ester (ISO-1) for different time periods, primed with MIF and stimulated with ANCA. Blank, untreated neutrophils; C5a + ANCA, neutrophils primed with C5a and stimulated with ANCA; 5–5 min, neutrophils pre-incubated with 5 μM ISO-1 for 5 min and then treated with C5a and ANCA; 10–5 min, neutrophils pre-incubated with 10 μM ISO-1 for 5 min and then treated with C5a and ANCA; 10–15 min, neutrophils pre-incubated with 10 μM ISO-1 for 15 min and then treated with C5a and ANCA. MFI, mean fluorescence intensity. **b** Respiratory burst in neutrophils (obtained from five healthy donors) under various experimental conditions, reflected by dihydrorhodamine-123 conversion into rhodamine-123. DMSO, dimethyl sulfoxide (control); IgG, normal immunoglobulin G; ISO-1, (S,R)3-(4-hydroxyphenyl)-4,5-dihydro-5-isoxazole acetic acid methyl ester (MIF antagonist); MFI, mean fluorescence intensity; MPO-ANCA-positive IgG, patient-derived myeloperoxidase-ANCA-positive immunoglobulin G; PR3-ANCA-positive IgG, patient-derived membrane proteinase-3-ANCA-positive immunoglobulin G. **c** Degranulation of neutrophils (obtained from five healthy donors) under various experimental conditions, determined from measurements of lactoferrin amounts in cell supernatants. DMSO, dimethyl sulfoxide (control); IgG, normal immunoglobulin G; ISO-1, (S,R)3-(4-hydroxyphenyl)-4,5-dihydro-5-isoxazole acetic acid methyl ester (MIF antagonist); MPO-ANCA-positive IgG, patient-derived myeloperoxidase-ANCA-positive immunoglobulin G; PR3-ANCA-positive IgG, patient-derived membrane proteinase-3-ANCA-positive immunoglobulin G. Bars represent mean ± standard deviation of measurements made in 5 independent experiments

MFI values for DHR oxidation were elevated in C5a-treated neutrophils administered MPO-ANCA- (902.6 ± 34.3 vs. 496.8 ± 25.4, *P* < 0.001) or PR3-ANCA- (899.8 ± 26.85 vs. 496.8 ± 25.4, *P* < 0.001) positive IgG, in comparison with non-treated cells (Fig. 4a). Pre-treatment with 5 μM or 10 μM ISO-1 (a MIF antagonist) for 5 min did not significantly reduce MFI values for DHR oxidation. However, pre-treatment with 10 μM ISO-1 for 15 min resulted in decreased production of oxygen radicals in C5a-treated neutrophils exposed to ANCA-positive IgG. Pre-treatment of C5a-primed cells with ISO-1 had no significant effect on mPR3 expression and MPO secretion (data not shown). In C5a-treated cells administered MPO-ANCA-positive IgG, ISO-1 reduced MFI values for DHR oxidation from 902.6 ± 34.32 (in the absence of ISO-1) to 677.6 ± 25.9 (*P* < 0.001) (Fig. 4b). In cells treated with PR3-ANCA-positive IgG, MFI values were decreased by ISO-1 from 899.8 ± 26.85 to 653.6 ± 36.52 (*P* < 0.001) (Fig. 4b).

Pretreatment with ISO-1 resulted in significantly decreased amounts of lactoferrin released in response to MPO-ANCA- or PR3-ANCA-positive IgG. Lactoferrin levels in cell supernatants rose from 602.4 ± 37.5 ng/ml in non-primed neutrophils to 1569.0 ± 48.2 ng/ml in C5a-treated cells administered MPO-ANCA-positive IgG (*P* < 0.001), and 1562.0 ± 73.9 ng/ml in C5a-treated cells exposed to PR3-ANCA-positive IgG (*P* < 0.001). However, upon pre-incubation with ISO-1, lactoferrin amounts in C5a-treated neutrophils were only slightly increased to 886.8 ± 37.1 ng/ml after treatment with MPO-ANCA-positive IgG (*P <* 0.001 vs. value in the absence of ISO-1) and 925.2 ± 30.0 ng/ml in cells treated with PR3-ANCA-positive IgG (*P <* 0.001 vs. value in the absence of ISO-1) (Fig. 4c).

### Supernatants of C5a-treated neutrophil cultures can cause unstimulated neutrophils to undergo ANCA-related respiratory burst and degranulation

**Fig. 5:**
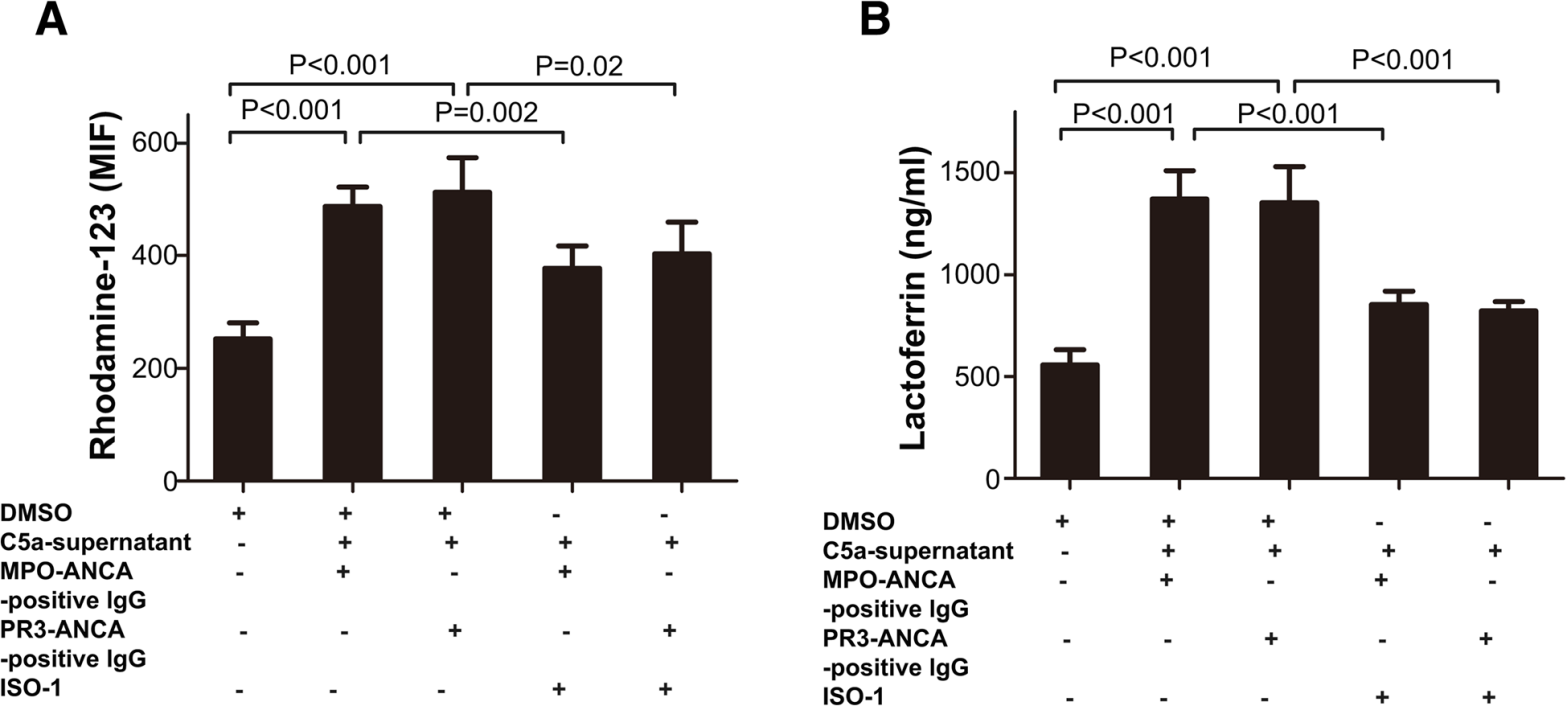
A macrophage migration inhibitory factor (MIF) antagonist inhibits respiratory burst and degranulation in neutrophils primed with culture supernatants from C5a-stimulated neutrophils and administered antineutrophil cytoplasmic antibody (ANCA). **a** Respiratory burst in neutrophils (obtained from five healthy donors) under various experimental conditions measured from dihydrorhodamine-123 conversion into rhodamine-123. DMSO, dimethyl sulfoxide (control); C5a-supernatant, supernatant from neutrophils primed with C5a; ISO-1, (S,R)3-(4-hydroxyphenyl)-4,5-dihydro-5-isoxazole acetic acid methyl ester (MIF antagonist); MFI, mean fluorescence intensity; MPO-ANCA-positive IgG, patient-derived myeloperoxidase-ANCA-positive immunoglobulin G; PR3-ANCA-positive IgG, patient-derived membrane proteinase-3-ANCA-positive immunoglobulin G. **b** Degranulation of neutrophils (obtained from five healthy donors) under various experimental conditions, determined from measurements of lactoferrin levels in culture supernatants. DMSO, dimethyl sulfoxide (control); C5a-supernatant, supernatant from neutrophils primed with C5a; ISO-1, (S,R)3-(4-hydroxyphenyl)-4,5-dihydro-5-isoxazole acetic acid methyl ester (MIF antagonist); MPO-ANCA-positive IgG, patient-derived myeloperoxidase-ANCA-positive immunoglobulin G; PR3-ANCA-positive IgG, patient-derived membrane proteinase-3-ANCA-positive immunoglobulin G. Bars represent mean ± standard deviation of measurements made in 5 independent experiments and donors

Next, neutrophils were primed using supernatants obtained from C5a-stimulated neutrophil cultures. In comparison with non-treated neutrophils, MFI values for DHR oxidation (i.e. oxygen radical production) were significantly higher in supernatant-treated cells administered MPO-ANCA- (487.0 ± 34.9 vs. 252.4 ± 28.5, *P* < 0.001) or PR3-ANCA- (512.2 ± 61.5 vs. 252.4 ± 28.5, *P* < 0.001) positive IgG. Pre-incubation of supernatant-treated neutrophils with ISO-1 resulted in decreased MFI values in cells treated with MPO-ANCA- (from 487.0 ± 34.9 in the absence of ISO-1 to 377.0 ± 39.9 in its presence, *P* < 0.001) or PR3-ANCA- (from 512.2 ± 61.5 in the absence of ISO-1 to 402.8 ± 55.9 in its presence, *P* < 0.001) positive IgG (Fig. 5a).

Pre-treatment with the MIF antagonist also suppressed lactoferrin release in response to MPO-ANCA- or PR3-ANCA-positive IgG. Lactoferrin amounts in supernatants rose from 555.0 ± 75.3 ng/ml in non-primed neutrophils to 1371.0 ± 140.6 ng/ml and 1352 ± 178.4 ng/ml in supernatant-treated cells administered MPO-ANCA- and PR3-ANCA-positive IgGs, respectively (both *P* < 0.001). In supernatant-primed neutrophils, pre-incubation with ISO-1 resulted in decreased lactoferrin amounts to 854.5 ± 65.6 ng/ml and 822.0 ± 45.73 ng/ml in cells treated with MPO-ANCA- and PR3-ANCA-positive IgGs, respectively (both *P* < 0.001 vs. respective values in the absence of ISO-1) (Fig. 5b).

## Discussion

Neutrophils are an important cell type involved in AAV [12, 13]. ANCAs probably have a direct role in the pathogenesis of AAVs by stimulating respiratory burst and degranulation in activated neutrophils [7–11]. Clinical and in vivo data indicate complement activation through the alternative pathway is also critical in AAV development [14–19, 44, 46, 47]. Among others, C5a can prime neutrophils for respiratory burst induced by ANCA [19]. Consistently, we previously demonstrated that translocation of ANCA antigens and ANCA-related respiratory burst in neutrophils are enhanced by recombinant C5a [45]. MIF is an immunomodulatory factor that regulates chemotaxis and survival in neutrophils [32, 33], and is involved in AAV [34, 35] and other autoimmune diseases [26–31]. Notably, elevated levels of plasma MIF were reported in patients with active AAV [37]. Furthermore, respiratory burst and degranulation by MIF-treated neutrophils are stimulated by MPO-ANCA- and PR3-ANCA-positive IgGs [37]. A previous study showed that C5a induces MIF production by neutrophils in sepsis [36], although the exact interaction between C5a and MIF as well as their roles in AAV remain mostly unknown. Therefore, the current study aimed to assess MIF’s role in the activation of C5a-primed neutrophils by ANCAs.

The present study indicated that neutrophils are an important source of MIF, and therefore may have a critical function in MIF release in pathologic conditions such as AAV, corroborating previous findings. MIF contributes to the pathogenesis of LPS-induced acute lung injury (ALI), and could be a therapeutic target in ALI [48]. Of note, MIF could also be a therapeutic target in a number of immune-related pathologies such as asthma [26], rheumatoid arthritis [27], chronic colitis [28], ulcerative colitis [29], glomerulonephritis [30], and lupus erythematosus [31], highlighting the possible clinical significance of the present study.

A previous report showed that patients with GPA or MPA have high MIF levels compared with those in remission [35]. Furthermore, a recent study by our group revealed that almost all patients with active MPA have high plasma levels of MIF [37]. Increased translocation of ANCA antigens is an important characteristic of neutrophils primed with MIF. ANCA-positive IgGs from patients with MPA can be used to further prime neutrophils and induce respiratory burst and degranulation. Therefore, we hypothesized that MIF promotes inflammation and disease activity in AAV.

MIF production after stimulation with recombinant human C5a and ANCA in neutrophils could be an important step in the initial stages of AAV. As shown above, ISO-1, a MIF antagonist, could significantly attenuate the effects of ANCA on neutrophils. Interestingly, C5a and MIF are proinflammatory factors that interact during ANCA-induced neutrophil activation. The above results are supported by a study of sepsis demonstrating that blockade or absence of the C5aR leads to a significant reduction in MIF biosynthesis [36].

Limitations of this study should be noted. First, direct measurements of MIF in cell culture supernatants were not performed. Secondly, residual C5a in cell supernatants may have contributed to the effects observed in supernatant-treated neutrophils. Thirdly, only one antagonist was used to inhibit MIF’s function, and the results should be confirmed using another antagonist or the siRNA technology. Fourthly, the molecular pathways underlying the interactions among C5a, MIF and ANCAs were not explored. Therefore, additional studies are required to confirm the current findings.

## Conclusions

Overall, the current findings suggest that MIF generated by C5a-treated neutrophils can further cause neutrophil activation. Furthermore, MIF blockage could reduce ANCA-induced activation of C5a-primed neutrophils. Thus, interaction between C5a and MIF may play a critical role during ANCA-induced activation of neutrophils. The findings presented here revealed a potential therapeutic candidate that could be targeted in order to control inflammatory injury in AAV.

## Data Availability

The datasets used and/or analysed during the current study are available from the corresponding author on reasonable request.

## Abbreviations

AAV: ANCA)-associated vasculitis
ALI: Acute lung injury
ANCA: Antineutrophil cytoplasmic antibody
BSA: Bovine serum albumin
DHR: Dihydrorhodamine
DMSO: Dimethyl sulfoxide
EGPA: Eosinophilic granulomatosis with polyangiitis
ELISA: Enzyme-linked immunosorbent assay
GPA: Granulomatosis with polyangiitis
HRP: Horseradish peroxidase
IgG: Immunoglobulin G
MFI: Mean fluorescence intensity
MIF: Migration inhibitory factor
MPA: Microscopic polyangiitis
MPO: Myeloperoxidase
mPR3: Membrane proteinase-3
PE: Phycoerythrin
